# Mpox Disease Severity Reduced in Intradermally MVA-BN Vaccinated Compared to Unvaccinated Patients in Sweden: A Retrospective 2024–2025 Observational Case-series

**DOI:** 10.1093/ofid/ofag337

**Published:** 2026-06-04

**Authors:** Catharina Missailidis, Anna Mia Ekström, Finn Filén, Andreas Jacks, Katarina Widgren, Magnus Gisslen, Klara Sondén, Kari Johansen

**Affiliations:** Department of Infectious Diseases/Venhälsan, Södersjukhuset, Stockholm, Sweden; Department of Clinical Science and Education, Karolinska Institutet Södersjukhuset, Stockholm, Sweden; Department of Infectious Diseases/Venhälsan, Södersjukhuset, Stockholm, Sweden; Department of Clinical Science and Education, Karolinska Institutet Södersjukhuset, Stockholm, Sweden; Department of Global Public Health, Karolinska Institutet, Stockholm, Sweden; Department of Infectious Diseases/Venhälsan, Södersjukhuset, Stockholm, Sweden; Department of Clinical Science and Education, Karolinska Institutet Södersjukhuset, Stockholm, Sweden; Department of Communicable Disease Prevention and Control Stockholm Region, Stockholm, Sweden; Department of Communicable Disease Prevention and Control Stockholm Region, Stockholm, Sweden; Department of Medicine, Karolinska Institutet, Stockholm, Sweden; Department of Infectious Diseases, Institute of Biomedicine, Sahlgrenska Academy at University of Gothenburg, Gothenburg, Sweden; Department of Infectious Diseases, Sahlgrenska University Hospital, Gothenburg, Sweden; Department of Medicine, Karolinska Institutet, Stockholm, Sweden; Department of Microbiology, Public Health Agency of Sweden, Solna, Sweden; Department of Global Public Health, Karolinska Institutet, Stockholm, Sweden; Department of Microbiology, Public Health Agency of Sweden, Solna, Sweden

**Keywords:** intradermal vaccination, HIV-1, mpox disease severity, MVA-BN vaccine

## Abstract

**Background:**

Modified Vaccinia Ankara–Bavarian Nordic vaccination (MVA-BN) prevents mpox, whether offered subcutaneously or intradermally. Intradermal administration has been implemented as a dose-sparing strategy, but evidence on durability of protection and long-term clinical impact is limited. We evaluated mpox severity over time in a limited number of mpox cases following primary intradermal MVA-BN vaccination.

**Methods:**

We conducted a retrospective case series of laboratory-confirmed mpox diagnosed between January 2024 and October 2025 at a major HIV and sexual health center in Stockholm, Sweden. Demographic and clinical data, vaccination history, and treatment outcomes were extracted from medical records. Disease severity was assessed using the validated Mpox Severity Score System.

**Results:**

Seventy-nine patients were included (median age, 40 years); 32 unvaccinated and 47 had prior vaccination with smallpox vaccine (n = 6), smallpox plus MVA-BN (n = 11), or MVA-BN only (n = 30). All MVA-BN doses were administered intradermally. Breakthrough infections occurred a median of 22 months after MVA-BN vaccination. Vaccinated individuals had significantly milder disease, with lower median severity score (5.0 vs 8.0; *P* < .0001), fewer lesions, more limited distribution, and less mucosal involvement. No MVA-BN-vaccinated patient developed complications with bacterial superinfection (*P* = .002), and analgesic requirements were reduced (*P* = .004). Disease severity showed a modest inverse correlation with time since vaccination (r = −0.42, *P* = .01). Similar attenuation was observed in vaccinated individuals with well-controlled HIV.

**Conclusions:**

Dose-sparing intradermal MVA-BN vaccination is associated with sustained reduction in mpox severity for up to 3 years. Continued vaccination of high-risk populations is supported, while prospective studies are needed to clarify long-term protection and optimize vaccination strategies.

## Teaser

Dose-sparing intradermal MVA-BN vaccination was associated with sustained reduction in mpox severity for up to 3 years. Vaccinated individuals experienced milder disease, fewer complications, and lower treatment needs, supporting continued vaccination of high-risk populations while studies evaluate long-term protection.

## BACKGROUND

The global mpox epidemic is ongoing, and vaccine shortages remain a significant challenge worldwide [1]. The World Health Organization recommends intradermal (ID) administration of the Modified Vaccinia Ankara–Bavarian Nordic (MVA-BN) vaccine at a fractional dose (0.1 mL per dose) in settings with limited vaccine supply and in outbreak settings, as an alternative to the standard 0.5-mL dose administered subcutaneously (SC) [2]. In response to high demand and restricted vaccine availability, Sweden adopted the ID vaccination strategy in accordance with guidance from the European Medicines Agency [3, 4]. This strategy has been maintained since August 2022 with few exceptions, making Sweden one of few countries with consistent long-term implementation of ID dosing, offered free of charge to targeted high-risk populations.

Although 2 SC administrations of MVA-BN have demonstrated vaccine effectiveness of approximately 66%–90% [5, 6] and case studies on breakthrough infections in patients predominantly MVA-BN vaccinated through SC or mixed SC/ID route observe reduced disease severity [5, 7], ID administration appears to confer comparable protection [8]. However, the long-term durability of mpox-specific immunity following ID vaccination—as well as its influence on the severity of subsequent breakthrough infections—remains unknown. Polymerase chain reaction-confirmed mpox infection is notifiable under the Swedish Communicable Diseases Act. To estimate the disease-moderating effects of primary MVA-BN ID vaccination over time, we conducted a retrospective case assessment of mpox disease severity in unvaccinated patients and MVA-BN-vaccinated patients with breakthrough infection during 2 mpox outbreak periods in 2024 and 2025. The study was conducted at a sexual health clinic for transgender individuals and cisgender men who have sex with men at Södersjukhuset, Stockholm, Sweden. The clinic is also an HIV center and serves as the regional MVA-BN vaccination center, where ongoing immunization has been offered since August 2022.

The primary objective of the study was to analyze the mpox disease-modifying effect of intradermal MVA-BN vaccination over time. The secondary objective was to analyze disease-modifying vaccine effects in smallpox-vaccinated patients with or without MVA-BN vaccine, and in people with HIV-1.

## METHOD

During the study period (1 January 2024–31 October 2025), a total of 95 mpox cases were reported to the Stockholm County Medical Officers. Of these, 80 patients (84%) were diagnosed and treated at the hospital Södersjukhuset. One patient did not fulfill inclusion criteria of more than 2 weeks’ duration from vaccination and was excluded leaving 79 patients eligible for assessment. Of the 79 patients, 32 individuals were unvaccinated and 47 were vaccinated according to documented MVA-BN administrations, stated or assumed childhood smallpox vaccination according to previous national vaccination program, and year of birth. In Sweden, smallpox vaccination stopped in 1976.

### Data

Demographic and clinical data including vaccination status, route and date of administered doses, prescribed pain medication, antibiotics, and bacterial culture results were retrospectively collected from medical records.

Disease severity was graded by 2 clinicians blinded to vaccination status using a validated mpox Severity Scoring System (mpox-SSS) [9]. Mpox-SSS is based on 7 clinical variables: total number of lesions, how many regions of the body have lesions, presence of confluent lesions, treatment for bacterial superinfection, number of affected mucosal sites, level of care required, and analgesia requirements. The severity score is calculated by summing the points assigned to each variable, resulting in a total score ranging from 0 to 23.

### Ethical Statement

Data were collected under the mandate of the Public Health Agency of Sweden for infectious disease surveillance and control, as defined by national legislation. Ethical approval was therefore not required. However, existing ethical approvals and informed consents (Dnr 2024-08356-02 and 2022-02235-01) for the clinics’ HIV preexposure prophylaxis (PrEP) cohort also cover sexually transmitted infection-related research, including mpox studies.

### Statistics

Univariate comparison of mpox severity score (mpox-SS) between unvaccinated and vaccinated individuals was performed using Mann-Whitney *U* test for continuous variables and Fisher exact test for categorical variables. Association between time from last administered MVA-BN vaccination and mpox-SS in vaccinated individuals was performed using Spearman correlation after testing for normal distribution. *P* < .05 was considered statistically significant. Analyses were performed with SPSS, version 29.0.1 (IBM, Armonk, New York, USA).

## RESULTS

The 79 mpox cases occurred in 2 major clusters: 22 cases in the second quarter (Q2) of 2024 and 47 cases between the first and second quarters (Q1-Q2) of 2025, with only sporadic cases reported during the remaining periods. All mpox infections were sexually acquired, with 63 infections contracted in Sweden and 16 abroad. Whole-genome sequencing by the Public Health Agency of Sweden (PHAS) identified two distinct lineages among the patients included in the study—F1 and F4—both belonging to monkeypox virus clade IIb.

The median age was 40 years (interquartile range [IQR]: 32–46, range 21–63 years) (Table 1). Of the 79 patients, 78 were cisgender male and 1 was a transgender woman. Most individuals (74/79) identified as gay or bisexual men who have sex with men. Twenty patients were living with virologically suppressed HIV, with a median CD4 count of 455 cells µL (IQR: 385–798, range 240–990) and median CD4 nadir of 337 (IQR: 244–480, range 140–630). Among the 59 HIV-negative individuals, 32 were using HIV-PrEP.

**Table 1.** Demographics of 79 Individuals With Incident Mpox January 2024-October 2025 in Stockholm, Sweden

Characteristics	All N = 79	Unvaccinated N = 32	Vaccinated N = 47		
			1 dose MVA-BN N = 5	2 doses MVA-BN N = 25	Smallpox vaccinated N = 17
**Age, median (range)**	40 (21–63)	34 (22–47)	33 (25–48)	41 (21–48)	51 (36–63)
**Cisgender male, n (%)**	78 (99)	31 (97)	5 (100)	25 (100)	17 (100)
**Transgender woman, n (%)**	1 (1)	1 (3)	0 (0)	0 (0)	0 (0)
**Men who have sex with men, n (%)**	74 (94)	27 (84)	5 (100)	25 (100)	17 (100)
**Heterosexual, n (%)**	5 (6)	5 (16)	0 (0)	0 (0)	0 (0)
**Has HIV, n (%)**	20 (25)	8 (25)	0 (0)	6 (21)	6 (35)
**HIV-PrEP user, n (%)**	32 (41)	4 (13)	2 (40)	18 (76)	8 (47)
**Non-HIV/non-HIV-PrEP user, n (%)**	27 (34)	20 (63)	3 (60)	1 (6)	3 (18)

Abbreviation: PrEP, preexposure prophylaxis.

Mpox infection was identified in 32 unvaccinated individuals: 8 (25%) were people with HIV, 4 (13%) were HIV-PrEP users, and 20 (63%) were neither HIV-positive nor using HIV-PrEP (Table 1).

Breakthrough infections following prior mpox vaccination were identified in 47 patients (Table 1). Among these 47 individuals, 30 were vaccinated exclusively with MVA-BN. Twenty-five (n = 25) had completed the 2-dose schedule, whereas 5 individuals received only a single dose. One individual did not complete the regimen because of an adverse reaction, and 4 received a single dose but were lost to follow-up. Seventeen (n = 17) individuals were smallpox vaccinated during childhood; 6 patients with smallpox vaccine only, 3 patients with smallpox vaccine and 1 dose MVA-BN administered in accordance with national mpox vaccination recommendation, and 8 patients with previous smallpox vaccine with 2 doses MVA-BN administered for unknown reasons.

All MVA-BN doses had been given through the ID route.

Patients with a history of smallpox vaccination were significantly older than those vaccinated with MVA-BN only (median age 51 years, IQR: 49–56, range 36–63 vs median age 41 years, IQR: 33–44, range 21–48; *P* < .001) and unvaccinated patients (median age 34 years, IQR: 30–40, range 22–47; *P* < .0001) (Table 1). Gender and sexual orientation were similarly distributed across the groups, with no significant differences in the proportion of individuals living with HIV. In contrast to the unvaccinated group, most vaccinated patients were HIV-PrEP users (4/32 13% vs 28/47, 60%; *P* < 0001), whereas non-HIV/non-HIV PrEP users were the dominating group among unvaccinated patients (20/32; 63%) (Table 1).

### mpox Severity Scoring

Primary analysis of mpox-SS in unvaccinated patients (n = 32) and patients vaccinated with MVA-BN vaccine only (n = 30) where a majority had been given 2 doses MVA-BV (n = 25); and secondary analysis of individuals with only one dose MVA-BV (n = 5), demonstrated significantly fewer number of lesions in vaccinated patients (*P* = .002 (Table 2). Vaccinated patients also had significantly less spread of lesions involving fewer parts of the body (*P* < .0001), which did not reach significant levels in patients with 1 dose (*P* = .12) (Table 2). Regardless, all patients with 1 MVA dose presented with lesions confined to 0 to 3 anatomical regions (5/5, 100%), whereas most unvaccinated patients had lesions confined to 4 or more regions (18/32, 56%) (Table 2). Moreover, vaccinated patients had significantly lower frequency of mucosal involvement (*P* = .04) (Table 2). The most affected mucosal sites in both groups were the anorectal mucosa and urethral mucosa, followed by the oral mucosa. No ocular involvement was detected in either group.

**Table 2.** mpox Severity Score Breakdown by Vaccination Status in 62 Individuals With Incident mpox, January 2024−October 2025, Stockholm, Sweden

Characteristics	Unvaccinated, N = 32	All MVA-BN Vaccinated N = 30	1 Dose MVA-BN N = 5	*P* Value unvaccinated: all MVA-BN Vaccinated^a,b^	*P* Value unvaccinated: 1 Dose MVA-BN^a,b^
**Lesion burden, number, n (%)**				.002	.97
0 (0 points)	1 (3)	4 (13)	0 (0)		
1–9 (1 point)	15 (47)	24 (80)	3 (60)		
10–99 (2 points)	16 (50)	2 (7)	2 (40)		
≥100 (3 points)	0 (0)	0 (0)	0 (0)		
**Anatomical regions involved^c^, n (%)**				<.0001	.12
0 (0 points)	2 (6)	4 (13)	0 (0)		
1–3 (1 point)	14 (44)	26 (87)	5 (100)		
4–6 (2 points)	8 (25)	0 (0)	0 (0)		
7–9 (3 points)	8 (25)	0 (0)	0 (0)		
10–12 (4 points)	0 (0)	0 (0)	0 (0)		
**Mucosal areas affected^d^, n (%)**				.04	.14
None (0 points)	7 (22)	13 (43)	2 (40)		
1 location (2 points)	24 (75)	17 (53)	3 (60)		
2 locations (3 points)	1 (3)	0 (0)	0 (0)		
≥3 locations (4 points)					
**Confluent lesion(s), diameter ≥2 cm** (2 points)	3 (9)	0 (0)	0 (0)	.24	1.0
**Bacterial superinfection** (2 points)**, n (%)**	9 (28)	0 (0)	0 (0)	.002	.31
**Level of care, n (%)**				.49	1.0
Outpatient (1 point)	30 (94)	30 (100)	5 (100)		
Inpatient, non-ICU related to Mpox (2 points)	2 (6)	0 (0)	0 (0)		
Inpatient, ICU related to Mpox (3 points)	0 (0)	0 (0)	0 (0)		
Death (4 points)	0 (0)	0 (0)	0 (0)		
**Need for analgesia/anesthetics, n (%)**				.004	.12
Outpatient, none (0 points)	6 (19)	11 (37)	2 (40)		
Outpatient, OTCs (1 point)	6 (19)	8 (25)	2 (40)		
Outpatient, prescribed (2 points)	18 (56)	6 (24)	1 (20)		
Inpatient, oral medications, hospitalized (3 points)	1 (3)	0 (0)	0 (0)		
Inpatient, IV medications, hospitalized (4 points)	1 (3)	0 (0)	0 (0)		
**mpox Severity Score (mpox-SS)**				<.0001	.003
Mean (SD)	8.1 (2.4)	4.8 (1.4)	5.0 (1.4)		
Median	8.0	5.0	6.0		
(IQR)	(6.3–9.0)	(4.0–6.0)	(3.5–6.0)		

^a^Mann-Whitney *U* test.

^b^Fisher exact test.

^c^Head/neck, chest/abdomen, back, groin/buttocks/anus, left arm, left hand, right arm, right hand, left leg, left foot, right leg, right foot.

^d^Anorectal, genital (urethral/vaginal), oropharyngeal, ocular lesions.

Abbreviations: ICU, intensive care unit; IQR, interquartile range; IV, intravenous; OTC, over the counter.

None of the MVA-BN–vaccinated patients developed recognized mpox-related complications such as confluent lesions, which were observed in 3 unvaccinated individuals (*P* = .24) (Table 2). Laboratory-confirmed or suspected bacterial superinfections caused by *Staphylococcus aureus* or *Streptococcus* groups A/C/G requiring antibiotic therapy were significantly less frequent among vaccinated patients (0/30 vs 9/32; *P* = .002) (Table 2). Although not statistically significant, only 3 patients—all unvaccinated—required hospitalization: 1 for pain management and 2 for bacterial superinfections (*P* = .49) (Table 2).

Vaccinated individuals required significantly less analgesic treatment overall (*P* = .004) (Table 2). In a subgroup analysis by type of analgesic therapy, prescriptions for topical anesthetics were significantly less frequent among vaccinated patients than among unvaccinated patients (4/30 vs 18/32; *P* = .002). In contrast, although opioids were prescribed less often to vaccinated patients, this difference did not reach statistical significance (4/30 vs 9/32, *P* = .22).

Finally, MVA-BN vaccination was associated with a significantly lower mpox-SS (median 5.0, IQR: 4.0–6.0 vs median 8.0, IQR: 6.3–9.0; *P* < .0001) (Table 2). A significantly lower severity score was also observed among individuals who had received a single dose of MVA-BN (median 6, IQR: 3.5–6.0; *P* = .003).

**Figure 1.**
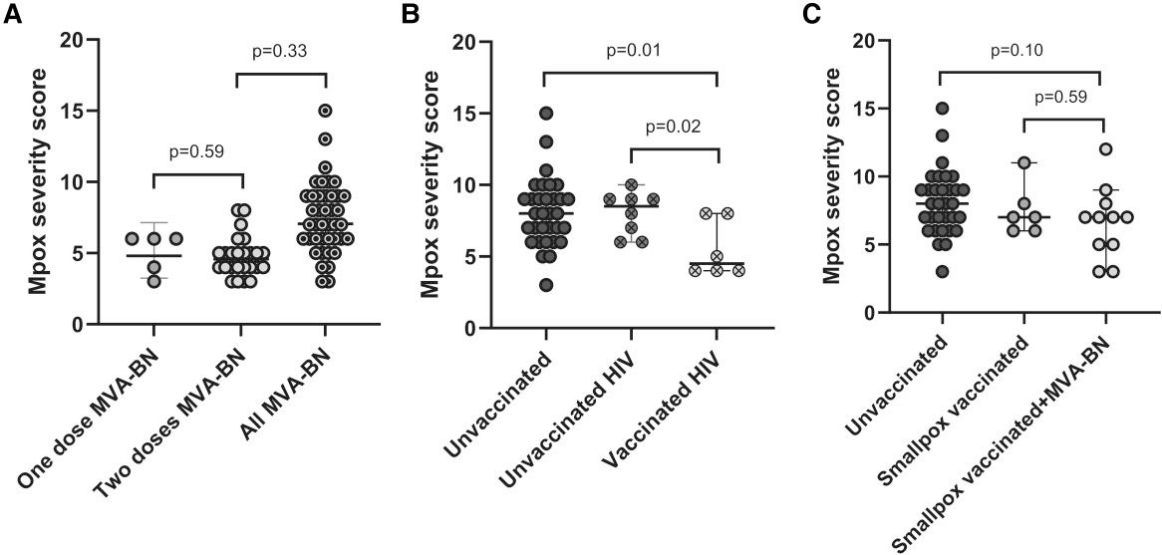
Subgroup analyses of mpox Severity Score with bars representing median and 95% confidence interval in: (*A*) Patients with 1 (n = 5) or 2 (n = 25) doses MVA-BN only, and patients with any dose MVA-BN including individuals with previous smallpox vaccination (n = 41). *B*, Unvaccinated patients (n = 32), of which 8 (n = 8) individuals have HIV and patients with HIV with 2 doses of MVA-BN (n = 6). *C*, Unvaccinated patients (n = 32) and patients with previous smallpox vaccine only (n = 6), and smallpox vaccine with any dose MVA-BN (n = 11). T test analyzed with Mann-Whitney *U* test.

Subgroup analyses found similar mpox-SS numbers in patients regardless of 1 dose MVA-BN (n = 5), 2 doses MVA-BN (n = 25), or previous smallpox followed by MVA-BN vaccination (n = 41) (Figure 1*A*). Unvaccinated people with HIV (n = 8), had similar mpox-SS as unvaccinated individuals without HIV (n = 24) (median 8.5, IQR: 6.3–9.0 vs median 8.0, IQR: 6.3–9.8; *P* = .96). Similarly, people with HIV vaccinated with 2 doses MVA-BN (n = 6) had mpox-SS as those without HIV (n = 19) (median 4.5, IQR: 4.0–8.0 vs median 5.0, IQR: 4.0–5.0; *P* = .40) and vaccination was significantly associated with lower mpox-SS (*P* = .02) (Figure 1*B*). Finally, no mpox-related complications were observed among patients with prior smallpox vaccination (n = 6) and those who had received smallpox vaccination followed by at least 1 dose of MVA-BN (n = 11). mpox-SS values in smallpox-vaccinated patients were numerically lower than in unvaccinated patients (median 7.0, IQR: 5.0–8.0 vs 8.0, IQR: 6.3–9.0) but did not reach statistical significance (Figure 1*C*).

**Figure 2.**
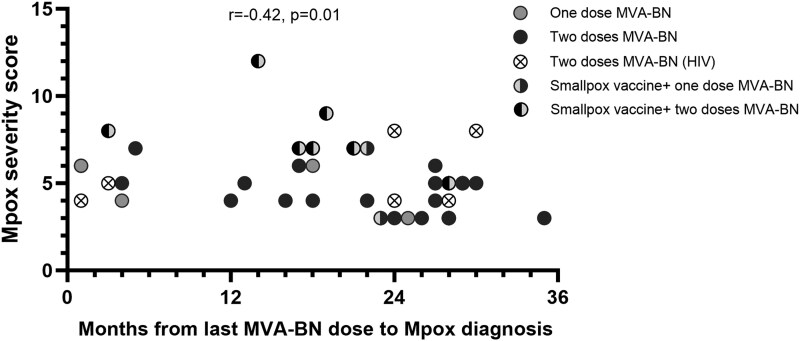
Time from vaccination and mpox-Severity Score in MVA-BN-vaccinated individuals. mpox-SS levels and time from last vaccination dose to disease in all patients with MVA-BN vaccination (n = 41): 5 (n = 5) patients with only 1-dose MVA-BN and 25 (n = 25) patients with 2 doses of MVA-BN, of which 6 patients have HIV, 3 (n = 3) patients with previous smallpox vaccine + 1 dose MVA-BN, and 8 (n = 8) patients with smallpox vaccine + 2 doses of MVA-BN. Rho (ρ) and *P* value derived from Spearmancorrelation analysis.

The median time from the last vaccine dose to breakthrough infection in patients with any dose MVA-BN (n = 41) was 22 months (IQR: 14–28, range 1–35) (Figure 2). Median time to breakthrough infection in MVA vaccinated only was shorter in those administered 1 dose (n = 5) than those administered 2 doses MVA-BN (n = 25) (median months 2, IQR: 1–22, range 1–22 vs 24, IQR: 15–28, range 1–35). Time to breakthrough infection in people with HIV with 2 doses MVA-BN (n = 5) were like those without HIV and 2 doses MVA-BN (n = 20) (median months 24, IQR: 13–29, range 1–33, vs 25, IQR: 14–28, range 3–35).

Disease severity in breakthrough infections, as determined by mpox-SS significantly decreased with time elapsed since the last vaccine dose (r = -0.42, *P* = .01) (Figure 2).

## DISCUSSION

This retrospective case series demonstrates that dose-sparing ID vaccination with MVA-BN is associated with sustained attenuation of mpox disease severity for up to 3 years following immunization with a median time of 22 months from vaccination to breakthrough infection.

Compared with unvaccinated individuals, vaccinated patients experienced fewer lesions, more limited spread of lesions, reduced mucosal involvement, fewer complications, and lower analgesic requirements, indicating clinically meaningful mitigation of disease.

Intradermal MVA-BN vaccination gave a reduction of median mpox-SS from 8.0 to 5.0. All MVA doses in this study were administered ID precluding head-to-head comparison with the SC route. However, a global case series assessing disease severity through mpox-SSS in breakthrough infection after 2 doses MVA-BN, the majority vaccinated SC and with similar demographics, found mpox-SS that closely matched our observation [7]. Similarly, mpox-SS in unvaccinated patients in our study matched those described in the mpox-SSS validation study [9].

Disease attenuation was most evident in individuals vaccinated with MVA-BN with no prior smallpox vaccination and who constituted the biggest group in this study. In contrast, those vaccinated against smallpox in childhood did not show a statistically significant reduction in severity, regardless of additional MVA-BN doses.

Smallpox vaccination has previously demonstrated significant reduction of advanced mpox symptoms [10]. It is unclear why we did not observe disease moderation in these patients, but interpretation is limited by small numbers and heterogeneous vaccination histories. The World Health Organization recommended cessation of routine smallpox vaccination in 1980; Sweden stopped smallpox vaccination 1976. Consequently, a history of childhood vaccination is inherently associated with older age as observed in this study where smallpox vaccinated patients were significantly older than unvaccinated (51 vs 34 years). This might confound results as age >45 years has been linked to more severe mpox symptoms [11].

In contrast to many mpox studies in which people with HIV constitute 40% to 50% of all cases, most patients in this study were HIV-PrEP users (41%), with only 25% with HIV. The cohort reflects the local epidemiology, with a high proportion of HIV-PrEP users and relatively few people with HIV. As with previous studies demonstrating similar disease severity in people with HIV with adequate CD 4 counts (>200–350 cells/L) as those without HIV [9], [12], we found comparable disease attenuation in vaccinated individuals with HIV—who were all virologically suppressed and immunologically stable with median CD 4 counts >455—and vaccinated individuals without HIV.

The observational nature of this case series precludes a definitive assessment of the relationship between time since vaccination and the risk of breakthrough infection as a surrogate marker for waning immunity. Although most breakthrough infections occurred at a median of 22 months following their last MVA-BN dose—an observation that could suggest partial decline in immunity over time—this pattern may alternatively reflect the temporal distribution of the vaccination campaign, which was concentrated in 2022 and early 2023 and remains ongoing. Notably, the protective effect of primary ID MVA-BN vaccination on disease severity remained consistent throughout the study period, with no evidence of diminished clinical impact with increasing time since immunization.

This analysis provides a granular clinical characterization of mpox breakthrough infections following ID MVA-BN vaccination in a high-volume clinical setting with extensive mpox expertise where vaccination status is well controlled. The analysis was supported by comprehensive clinical and microbiological data and systematic chart review. The cohort reflects a real-world vaccination program with follow-up of up to 3 years in a high-risk population with high vaccine uptake, thereby enhancing the public health relevance and external validity of the findings.

However, the retrospective observational design with mpox-SS components depending on information provided from medical chart information introduces an inherent risk of bias weakening the strength of the analyses. Also, the limited case numbers restrict causal inference and limit adjustment for potential confounders. Importantly, the data set lacks comparative analyses on disease severity in subcutaneously vaccinated breakthrough cases. Furthermore, it is a single-center study and although the vast majority of all registered Mpox cases in the Stockholm region are handled at the center, this may limit generalizability because the center primarily caters to transgender individuals and cisgender men who have sex with men. It may also introduce screening bias whereby standardized monitoring of HIV-PrEP users and HIV patients with high vaccination coverage may lead to the detection of more asymptomatic or mild cases in these groups that might otherwise have gone unnoticed, thereby confounding the observed vaccination effect in comparison with unvaccinated individuals.

## CONCLUSION

Dose-sparing intradermal MVA-BN vaccination is associated with clinically significant reduction in mpox disease severity for up to 3 years in those who developed breakthrough infection. Vaccination should remain a prioritized strategy for individuals at elevated risk since the mpox epidemic is still ongoing globally. Prospective, comparative studies are needed to define the long-term durability of protection and to directly compare ID and SC vaccination strategies.
